# Mechanistic rationale for MCL1 inhibition during androgen deprivation therapy

**DOI:** 10.18632/oncotarget.3368

**Published:** 2015-01-14

**Authors:** Frédéric R. Santer, Holger H. H. Erb, Su Jung Oh, Florian Handle, Gertrud E. Feiersinger, Birgit Luef, Huajie Bu, Georg Schäfer, Christian Ploner, Martina Egger, Jayant K. Rane, Norman J. Maitland, Helmut Klocker, Iris E. Eder, Zoran Culig

**Affiliations:** ^1^ Medical University of Innsbruck, Department of Urology, Division of Experimental Urology, Innsbruck, Austria; ^2^ Yorkshire Cancer Research Unit, University of York, York, United Kingdom; ^3^ Medical University of Innsbruck, Department of Plastic, Reconstructive & Aesthetic Surgery, Innsbruck, Austria

**Keywords:** endocrine therapy, cell cycle arrest, cell death, treatment resistance, BCL2 family

## Abstract

Androgen deprivation therapy induces apoptosis or cell cycle arrest in prostate cancer (PCa) cells. Here we set out to analyze whether MCL1, a known mediator of chemotherapy resistance regulates the cellular response to androgen withdrawal. Analysis of MCL1 protein and mRNA expression in PCa tissue and primary cell culture specimens of luminal and basal origin, respectively, reveals higher expression in cancerous tissue compared to benign origin. Using PCa cellular models *in vitro* and *in vivo* we show that MCL1 expression is upregulated in androgen-deprived PCa cells. Regulation of MCL1 through the AR signaling axis is indirectly mediated via a cell cycle-dependent mechanism. Using constructs downregulating or overexpressing MCL1 we demonstrate that expression of MCL1 prevents induction of apoptosis when PCa cells are grown under steroid-deprived conditions. The BH3-mimetic Obatoclax induces apoptosis and decreases MCL1 expression in androgen-sensitive PCa cells, while castration-resistant PCa cells are less sensitive and react with an upregulation of MCL1 expression. Synergistic effects of Obatoclax with androgen receptor inactivation can be observed. Moreover, clonogenicity of primary basal PCa cells is efficiently inhibited by Obatoclax. Altogether, our results suggest that MCL1 is a key molecule deciding over the fate of PCa cells upon inactivation of androgen receptor signaling.

## INTRODUCTION

Androgen deprivation therapy (ADT) is a first-line therapy for locally advanced and metastatic prostate cancer (PCa). This includes the use of Gonadotropin-releasing hormone (Luteinizing-hormone releasing hormone) agonists and antagonists suppressing the production of testosterone, and non-steroidal anti-androgens (e.g. Bicalutamide (Casodex^TM^)) that inhibit activation of the androgen receptor (AR) by competing with its natural ligand dihydrotestosterone [1]. Despite initial success, most patients experience progression within 12 to 33 months to a more aggressive phenotype termed castration-resistant PCa (CRPCa) concurrent with a reactivation of the AR signaling axis. ADT induces shrinkage of the tumor or stops its growth. On the cellular level, the consequences of ADT on malignant prostate epithelial cells are induction of apoptosis or cell cycle arrest in G_1_ phase [2]. Obviously, cells reacting with a cell cycle arrest and unable to induce apoptosis may be at the basis of development of castration resistance. Hence, in order to improve the efficiency of ADT, combination therapies are warranted where the ADT-additive therapy targets the G_1_ cell cycle-arrested PCa cells.

We hypothesized that the molecular difference between the apoptosis- and cell cycle arrest- inducing effects of ADT could be found among members of the BCL2 protein family, which act as a rheostat regulating the balance between survival and apoptosis [3]. Pro-apoptotic proteins of this family commit cells to programmed death by permeabilising the outer mitochondrial membrane followed by cytochrome C release. MCL1 is a pro-survival member of the BCL2 family and prevents activation of pro-apoptotic BCL2-homology (BH)3-only proteins and the effector BAK1 [4]. MCL1 expression is induced by a number of cytokines and growth factors and is tightly regulated at transcriptional, post-transcriptional and post-translational levels [5]. High protein expression levels and somatic copy-number amplifications of the *MCL1* gene have been found in several cancer types [6]. MCL1 has superior apoptosis-inhibitory functions compared to other BCL2 family members [7]. It confers multi-drug resistance [8] and, moreover, resistance to ABT-737, a BH3-mimetic inhibiting anti-apoptotic BCL2 family members with the exception of MCL1 [9]. In contrast, Obatoclax (GX15-070), which also targets MCL1, can overcome ABT-737-mediated resistance [10]. Obatoclax has been assessed in clinical studies in combinatorial approaches with existing therapies [11-13].

Here, we demonstrate that high expression of MCL1 promotes the survival of steroid-deprived and cell cycle-arrested PCa cells. Our data suggests that inhibition of MCL1 could improve currently used ADT protocols by targeting the G_1_ phase-arrested cell population.

## RESULTS

### Increased expression of MCL1 in malignant compared to benign areas in prostate tissue specimens

**Figure 1:**
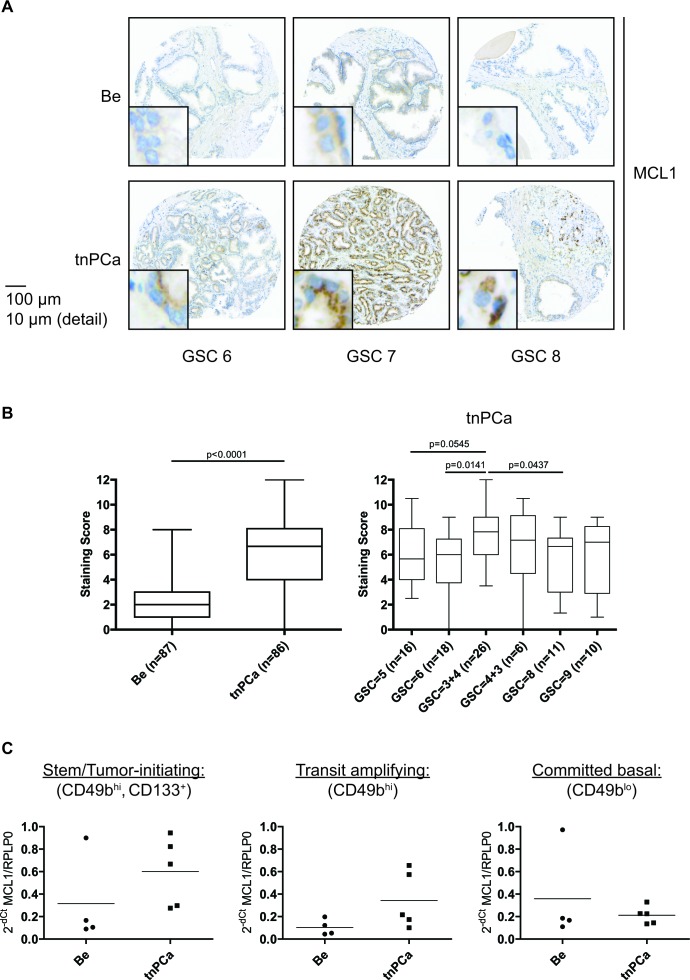
Increased expression of MCL1 in malignant areas of treatment-naïve prostate tissue (A, B) Immunohistochemistry for MCL1 expression was performed on a TMA arranged with samples from treatment-naïve PCa (tnPCa) patients undergoing radical prostatectomy. Stainings from cancerous areas of 86 patients and adjacent benign areas of 87 patients were evaluable. (A) Representative images of positive MCL1 staining from paired tissue specimens of malignant areas (tnPCa) with Gleason Score (GSC) 6, 7 and 8, and adjacent benign areas (Be) are shown. (B) MCL1 staining was evaluated by an uropathologist using the quickscore system and the resulting staining scores are illustrated in box and whiskers graphs. (C) MCL1 mRNA expression was determined in primary basal benign and malignant cells after sorting into stem/tumor-initiating cell (SC/TIC, CD133+, CD49bhi), transit amplifying (TA, CD49bhi) and committed basal (CB, CD49blo) populations. Benign (Be, n=4) and malignant (tnPCa, n=5) primary basal cells were isolated from tissue specimens of treatment-naïve PCa patients undergoing radical prostatectomy. All samples were grown in cell culture *in vitro* and SC/TIC, TA and CB subpopulations were isolated as previously described [43]. Samples were subjected to qRT-PCR analysis for MCL1. MCL1 mRNA expression was normalized to the housekeeper RPLP0 and is expressed as 2^−dCt^.

In order to assess expression of MCL1 in prostatic tissue and to validate MCL1 as a potential target for treatment of PCa we performed immunohistochemistry on tissue specimens from treatment-naïve prostate cancer (tnPCa) patients who underwent radical prostatectomy (Fig. 1A). A significantly increased staining score of cytoplasm-localized MCL1 could be observed in malignant compared to adjacent benign areas (Fig. 1A, detail views; Fig. 1B, left). However, we could not observe a positive correlation of MCL1 expression with Gleason score (Fig. 1B, right). Additionally, we analyzed MCL1 mRNA expression in primary basal, androgen-independent [14] cells grown from benign and malignant biopsies from tnPCa gained after radical prostatectomy (Fig. 1C). To determine whether MCL1 is differentially expressed with increasing cell differentiation, we separated committed basal (CB, CD49b^lo^) from transit amplifying cells (TA, CD49b^hi^) based on their potential to attach to type I collagen. Consequently, stem/tumor-initiating cells (SC/TIC) were isolated from the TA population by making use of their CD133 expression [15]. MCL1 mRNA expression was then measured by qRT-PCR on isolated cell populations. We found that MCL1 mRNA is increasingly expressed in malignant compared to benign samples in SC/TIC and TA populations. Intriguingly, TIC showed highest increase of MCL1 mRNA expression levels compared to benign SC, which could point to increased apoptotic resistance of TIC. Altogether, this showed that MCL1 expression is increased in basal and luminal prostatic compartments of cancerous compared to benign origin.

### Activation of the AR signaling axis leads to decreased MCL1 expression levels

**Figure 2:**
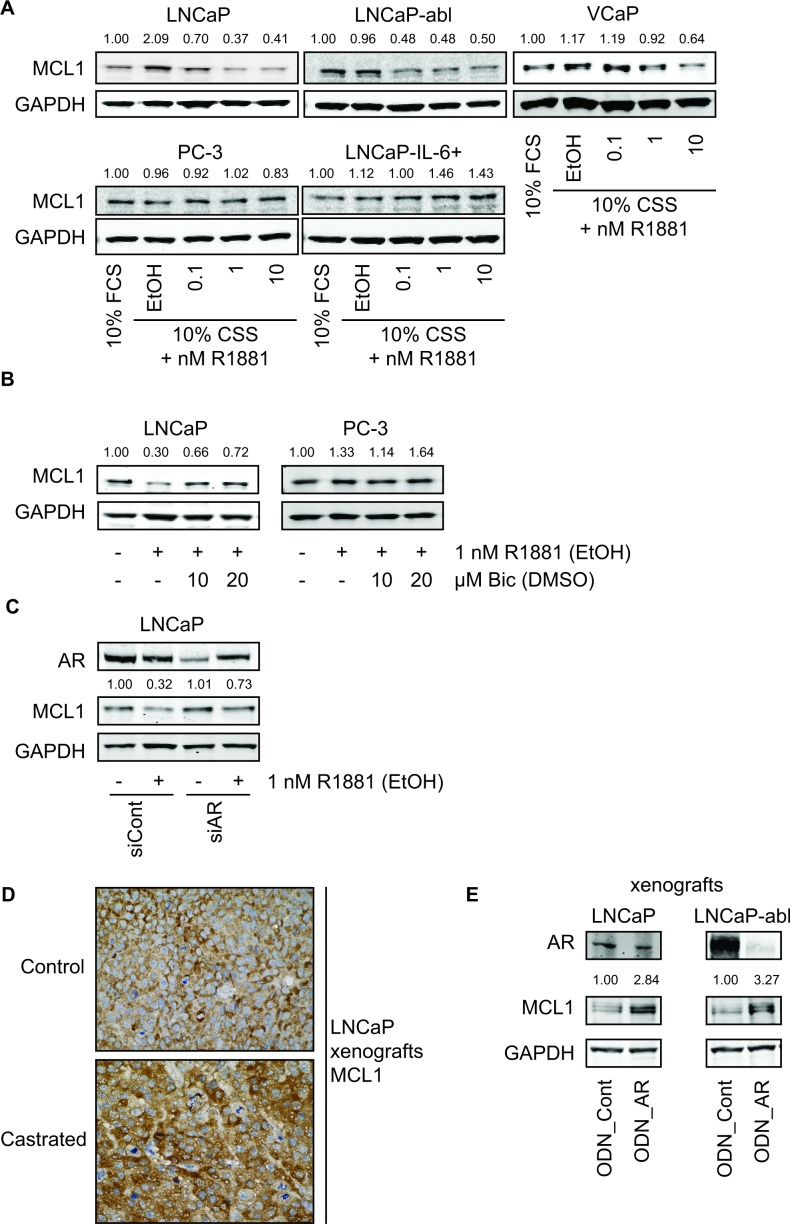

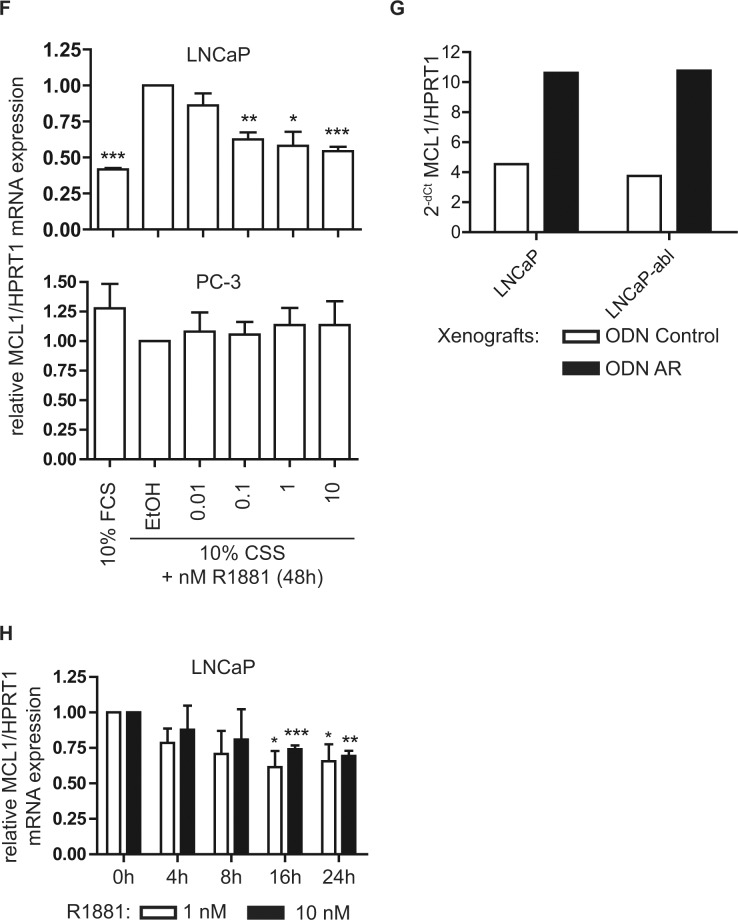
Androgenic regulation of MCL1 is dependent on functional AR and is a transcriptional mechanism (A) Activation of the AR signaling axis through R1881 decreases MCL1 protein expression. PCa cell lines were steroid-deprived for ≥48 h using medium supplemented with 10% CSS and then treated for additional 48 h in medium supplemented with 10% FCS or in medium supplemented with 10% CSS and increasing concentrations of R1881 or vehicle (EtOH), as indicated. (B) Inhibition of AR by Bicalutamide counteracts the effects of R1881 on MCL1 expression. LNCaP and PC-3 were steroid-deprived for ≥48 h using medium supplemented with 10% CSS and then treated for additional 48 h in medium supplemented with 10% CSS and 1 nM R1881 or vehicle (EtOH) with 10 or 20 μM Bicalutamide (Bic) or vehicle (DMSO), as indicated. (C) Downregulation of AR by siRNA counteracts the effects of R1881 on MCL1 expression. LNCaP were steroid-deprived for ≥48 h using medium supplemented with 10% CSS and then transfected with 30 nM siRNA targeting AR (siAR) or control siRNA (siCont) and treated with 1 nM R1881 or vehicle (EtOH) for 48 h, as indicated. (D) MCL1 expression is increased in LNCaP xenografts grown in castrated mice. LNCaP were implanted subcutaneously in both flanks of BALB/c nu/nu mice. When tumors became palpable animals were castrated (6 animals, 12 tumors) or left intact (6 animals, 12 tumors). Tumors were explanted four weeks after castration and immunohistochemistry for MCL1 was performed on formalin-fixed, paraffin-embedded sections. Representative stainings for MCL1 of LNCaP xenografts from intact and castrated mice are shown. Statistical analysis of MCL1 intensities of intact vs. castrated animals showed mean intensity (−/+ SD) of 1.92 (−/+ 0.29) vs. 2.25 (−/+ 0.62); n=12; p=0.1026, respectively. (E) MCL1 expression is increased in LNCaP and LNCaP-abl xenografts after downregulation of AR by oligodeoxynucleotides (ODN). LNCaP and LNCaP-abl were injected subcutaneously in intact or castrated BALB/c nu/nu mice, respectively. When tumors became palpable intraperitoneal injections with 5 mg/kg control oligodeoxynucleotides (ODN_Cont, n=6) or 5 mg/kg ODN targeting the AR (ODN_AR, n=6) were performed three times in the first week and twice in the following three weeks. After four weeks under this treatment protein was extracted from an explanted tumor from animals bearing LNCaP or LNCaP-abl xenografts transduced with either ODN_Cont or ODN_AR. (A-C, E) Protein expression was determined by immunoblotting using antibodies for AR, MCL1 and GAPDH, as indicated. Representative immunoblots are shown. Numbers indicate relative ratios of densitometrical analysis of MCL1 divided by the reference protein GAPDH. (F) MCL1 mRNA expression is regulated through the action of androgens. LNCaP and PC-3 cells were steroid-deprived for ≥48 h using medium supplemented with 10% CSS and then treated for additional 48 h with medium supplemented with 10% FCS or in medium supplemented with 10% CSS and increasing concentrations of R1881 or vehicle (EtOH), as indicated. (G) MCL1 mRNA is increased in LNCaP and LNCaP-abl xenografts transduced with ODN targeting the AR. From the animal experiment performed as described in (E) mRNA was extracted from an explanted tumor from animals bearing LNCaP or LNCaP-abl xenografts receiving injections with control ODN (ODN_Cont) or AR ODN. (H) Androgenic regulation of MCL1 is significantly apparent after 16 h of incubation with R1881. LNCaP were steroid-deprived for ≥48 h using medium supplemented with 10% CSS and then treated in medium supplemented with 10% CSS and increasing concentrations of 1 or 10 nM R1881 or vehicle (EtOH) for different time points, as indicated. (F-H) MCL1 mRNA was quantified using qRT-PCR, normalized to the housekeeper HPRT1 and results are illustrated as relative mean ± SEM, n≥3 (F, H) or in absolute values as 2^−dCt^ (G). Statistical significances were calculated against EtOH-treated sample and encoded as follows: * p<0.05; ** p<0.01; *** p<0.001.

Next, we analyzed the role of AR signaling and androgen deprivation on MCL1 expression levels using established cell culture models of PCa. Surprisingly, AR inactivation through steroid deprivation (using 10% charcoal-stripped serum, CSS) caused an increase of MCL1 in LNCaP and to a lesser extent in VCaP cells compared to normal growth conditions (10 % fetal calf serum, FCS) (Fig. 2A). This effect was lost in LNCaP-abl, a derivative of the LNCaP cell line that has adapted to steroid-deprived conditions but has retained androgen sensitivity [16]. On the other hand, treatment with the synthetic androgen R1881 for 48 h decreased MCL1 expression in a concentration-dependent manner in the androgen-sensitive cell lines LNCaP, LNCaP-abl and VCaP. MCL1 expression in the AR-negative cell lines PC-3 and LNCaP-IL-6+ [17] did not decrease upon R1881 treatment. To confirm the involvement of AR in the regulation of MCL1, AR activity was inhibited by the anti-androgen Bicalutamide (Fig. 2B) or by knocking down its expression by means of siRNA specific for AR (Fig. 2C). In both cases decreased MCL1 expression through the action of R1881 could be antagonized, while AR-negative PC-3 were unaffected by Bicalutamide treatment. R1881 treatment was able to partly counteract the siRNA-mediated downregulation of AR. Analysis of explants from an *in vivo* experiment, where LNCaP were xenografted subcutaneously in male nude BALB/c mice, showed increased staining for MCL1 when animals were castrated compared to intact mice (Fig. 2D). Similarly, when intact animals bearing LNCaP or castrated animals bearing LNCaP-abl xenografts received injections with oligodeoxynucleotides (ODN) targeting AR, increased expression levels of MCL1 were observable (Fig. 2E).

To address the question whether androgenic repression of MCL1 is a transcriptional or post-translational mechanism, we measured MCL1 mRNA levels 48 h after treatment with R1881 (Fig. 2F) and protein levels after knock-down of three known E3-ligases APC/C^CDC20^, SCF^FBXW7^ and HUWE1 [18] (Fig. S1A). MCL1 mRNA was decreased in a dose-dependent manner through R1881 in LNCaP, while AR-negative PC-3 cells were unaffected. On the other hand, none of the three E3-ligases was able to antagonize the effect of R1881 on MCL1 protein expression. Similarly, the increased MCL1 protein expression seen in LNCaP and LNCaP-abl xenografts after knock-down of AR (Fig. 2E) was mediated by increased MCL1 mRNA expression (Fig. 2G). Time course experiments following addition of R1881 showed that androgenic repression of MCL1 was significant after 16 h at mRNA levels (Fig. 2H) and after 24 h at the protein level (Fig. S1B). Altogether, we concluded that androgenic signaling through the AR axis is able to repress MCL1 protein and mRNA expression.

### Androgenic repression of MCL1 is a cell cycle dependent mechanism

**Figure 3:**
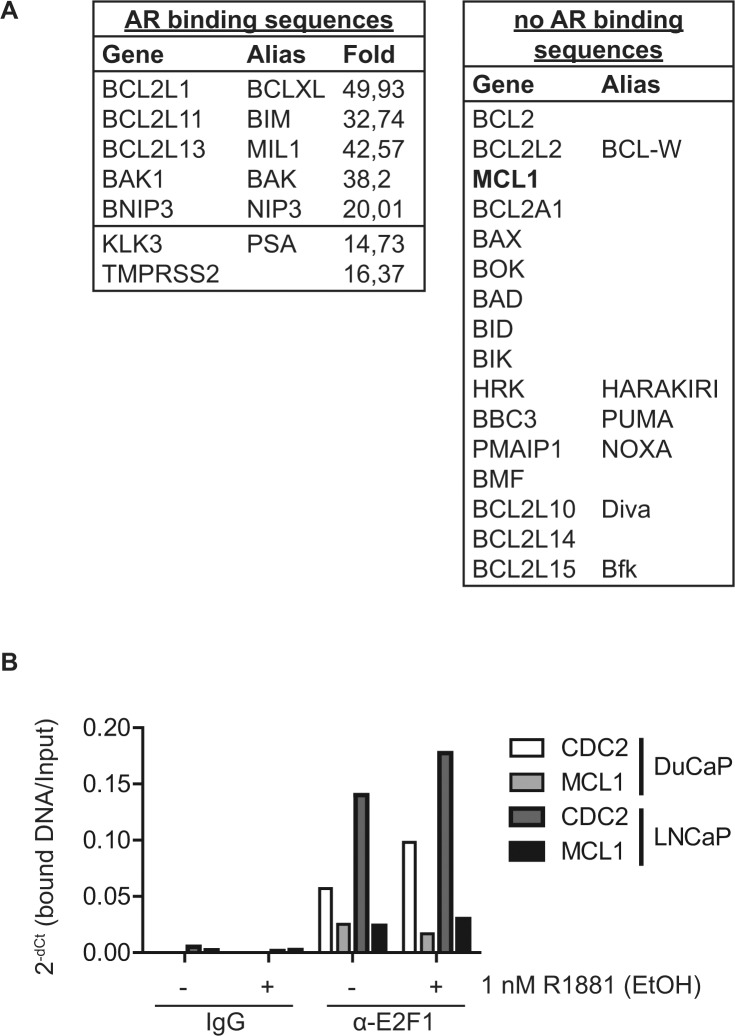

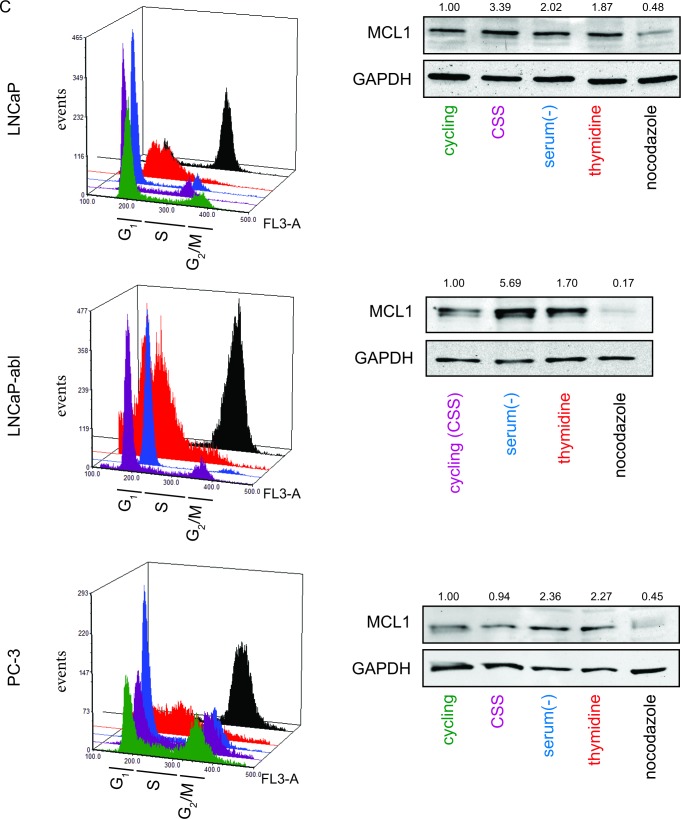
MCL1 is a cell cycle regulated protein (A) The MCL1 gene locus does not contain AR binding sites. Chromatin immunoprecipitation (ChIP) was performed on DuCaP cells treated for 1 h with 1 nM R1881 or vehicle (EtOH) using anti-AR antibodies [19]. DNA bound to the precipitated AR complex was subjected to deep sequencing (ChIP-seq). The resulting gene list was searched for members of the BCL2 family and results are depicted in the tables. The fold change indicates the enrichment of AR bound to the respective sequence of R1881-treated versus vehicle-treated samples. Only the top fold regulation is shown. (B) The cell cycle regulatory transcription factor E2F1 binds to the promoter region of the MCL1 gene. DuCaP and LNCaP cells were steroid-deprived for ≥48 h using medium supplemented with 10% CSS and then treated for additional 24 h in medium supplemented with 10% CSS and 1 nM R1881 or vehicle (EtOH). ChIP was performed using antibodies against E2F1 or control isotype IgG. DNA bound by E2F1 or control IgG was quantified using primers against promoter regions of MCL1 or CDC2. Values were normalized to input taken before IP and are depicted as 2^−dCt^. (C) MCL1 is increasingly expressed in G1 and S phases, while decreased during G2/M. Various PCa cells were arrested in different cell cycle phases through complete serum deprivation (G_1_, blue), excess of thymidine (G_1_/S, red) and nocodazole treatment (G_2_/M, black). Additionally, normal proliferating cells (cycling, green) and steroid-deprived cells (10% CSS, violet) are shown. LNCaP-abl cells actively proliferate in steroid-deprived medium. Histogram blots illustrate the distribution of cell cycle phases which was assessed by the DNA stain propidium iodide using flow cytometry. Representative immunoblots for MCL1 and GAPDH are shown. Numbers indicate relative ratios of densitometrical analysis of MCL1 divided by the reference protein GAPDH.

Since decreased expression of MCL1 at the mRNA level was observable after 16 h R1881 treatment (Fig. 2H), we hypothesized that this regulation is not directly mediated by AR but rather indirectly by another AR-stimulated transcription factor. Indeed, in a genome-wide chromatin-immunoprecipitation experiment coupled with deep sequencing (ChIP-seq) in DuCaP cells [19] we were not able to identify AR binding sites neighboring or within the MCL1 gene, in contrast to other known AR targets and, surprisingly, other BCL2 family members (Fig. 3A and S2A).

Activated AR is a potent stimulator of cell cycle progression from G_1_ to S phase in cancer cells through the Cyclin D1/retinoblastoma/E2F1 axis [2]. Previously, the cell cycle controller E2F1 was found to act as a repressor of the MCL1 promoter [20]. Thus, AR might repress MCL1 expression indirectly via E2F1 binding to the MCL1 promoter. We confirmed E2F1-binding to the MCL1 promoter in a ChIP experiment (Fig. 3B). E2F1 binding intensity to the MCL1 promoter was comparable to the known E2F1 target CDC2, however only mildly altered by the addition of R1881 implying that other co-factors regulating E2F1 transcriptional activity might be involved.

Next, we analyzed whether increased expression levels of MCL1 through steroid-depletion could be mediated via a G_1_ cell cycle arrest caused by the inactivation of AR. To this end, we arrested PCa cells in G_1_, G_1_/S and G_2_/M by serum withdrawal, thymidine excess and nocodazole treatment, respectively (Fig. 3C and S2B). Immunoblotting for MCL1 showed increased levels in G_1_ and G_1_/S phases, while, as previously published [21], decreased levels were detected after nocodazole treatment. This effect was observable in AR-positive and –negative cell lines showing that MCL1 levels could be increased by G_1_ cell cycle arrest independently of the AR status. In other words, androgenic regulation of MCL1 is an indirect mechanism through AR inactivation and consequent G_1_ cell cycle arrest.

### MCL1 protects PCa cells from undergoing apoptosis under steroid-depleted conditions

**Figure 4:**
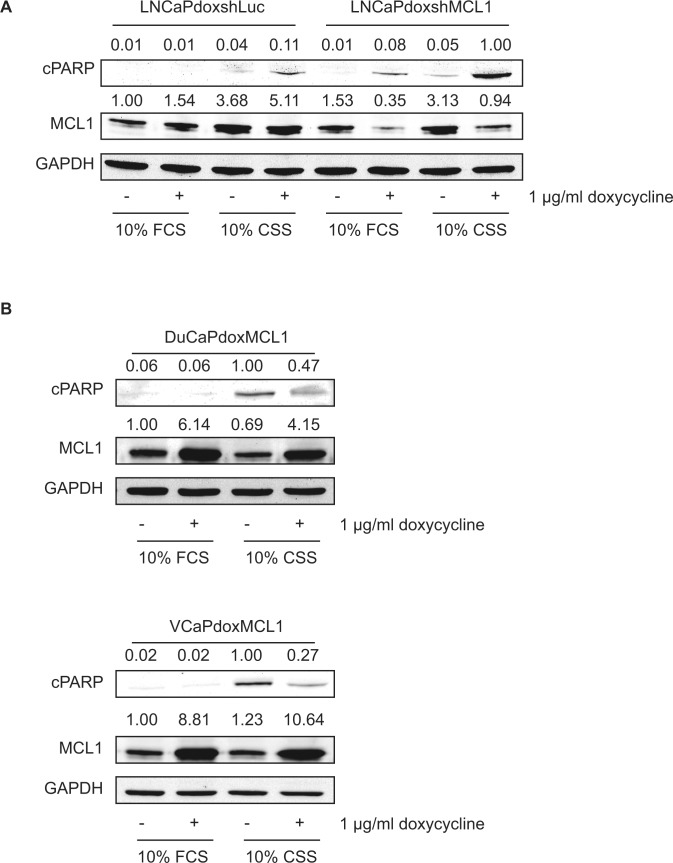
MCL1 protects cells from undergoing apoptosis under steroid-deprived conditions (A) Knockdown of MCL1 in LNCaP cells leads to increased apoptosis under steroid-deprived conditions. LNCaP cells were stably transduced with doxycycline-regulated short hairpin (sh) constructs targeting MCL1 (LNCaPdoxshMCL1) or Luciferase (control, LNCaPdoxshLuc). Both cell lines were grown for 72h under full medium conditions (10% FCS) or under steroid-deprived conditions (10% CSS) and expression of sh constructs was induced by addition of 1 μg/ml doxycycline, as depicted. (B) Overexpression of MCL1 rescues DuCaP and VCaP cells partially from undergoing apoptosis when grown in steroid-deprived conditions. DuCaP and VCaP were stably transduced with a doxycycline-regulated overexpression plasmid for MCL1 generating DuCaPdoxMCL1 and VCaPdoxMCL1. Both cell lines were grown for 40h under full medium conditions (10% FCS) or under steroid-deprived conditions (10% CSS) and overexpression of MCL1 was induced by addition of 1 μg/ml doxycycline. (A, B) Samples were analyzed by immunoblotting using antibodies against MCL1, cleaved PARP (cPARP, as an indicator for apoptosis) and GAPDH. Representative immunoblots are shown. Numbers indicate relative ratios of densitometrical analysis of MCL1 or cPARP divided by the reference protein GAPDH, respectively.

Subsequently, we analyzed the functional role of MCL1 upregulation after AR inactivation and G_1_ cell cycle arrest. LNCaP cells showed increased expression of MCL1 under steroid-deprived conditions but only a mild induction of apoptosis (Fig. 4A). To test the hypothesis that upregulated MCL1 protects LNCaP from undergoing apoptosis during steroid ablation we made use of a doxycycline-inducible short hairpin construct to downregulate MCL1 (shMCL1). While either steroid deprivation, or MCL1 downregulation under full growth conditions (10% FCS) induced apoptosis only weakly, the combination of steroid deprivation and MCL1 downregulation resulted in a strong induction of apoptosis. In contrast, DuCaP and VCaP reacted with a strong apoptotic response to steroid ablation but only mild upregulation of MCL1 (Fig. 4B). Overexpression of MCL1 using doxycycline-inducible overexpression of MCL1 could partly rescue DuCaP and VCaP cells from undergoing apoptosis when grown in CSS-containing medium. In summary, our data shows that MCL1 acts as a pro-survival molecule under steroid-ablated conditions and, hence, targeting MCL1 might be a valuable addition to a steroid deprivation protocol.

### Induction of apoptosis after pharmacological inhibition of MCL1 is dependent on the progression state of PCa cells

**Figure 5:**
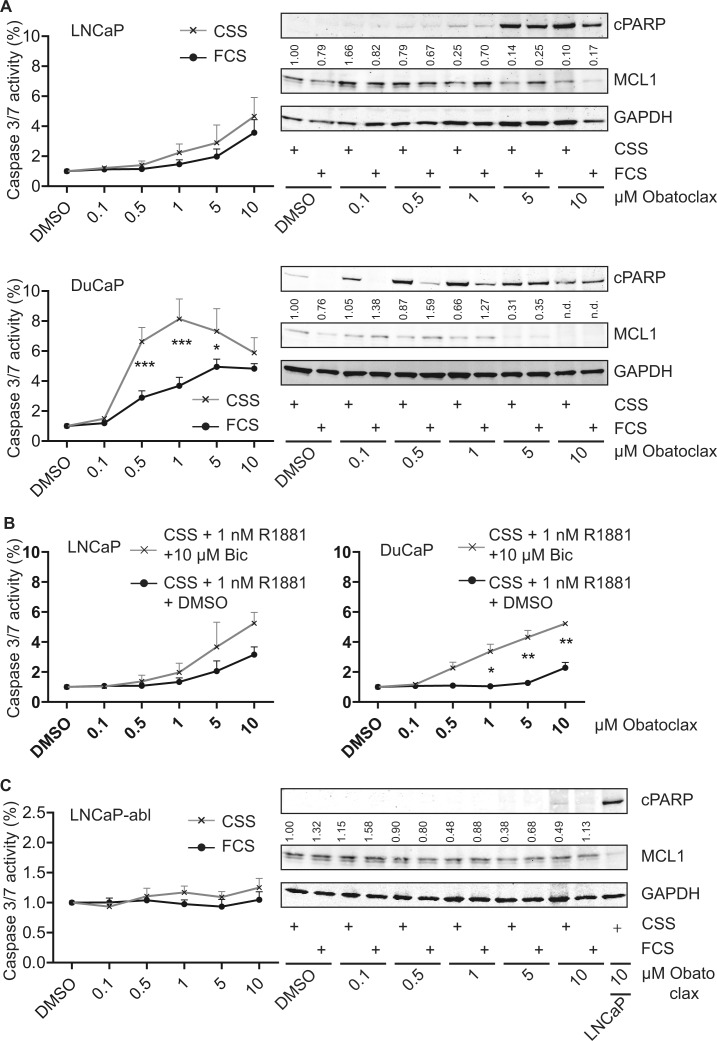

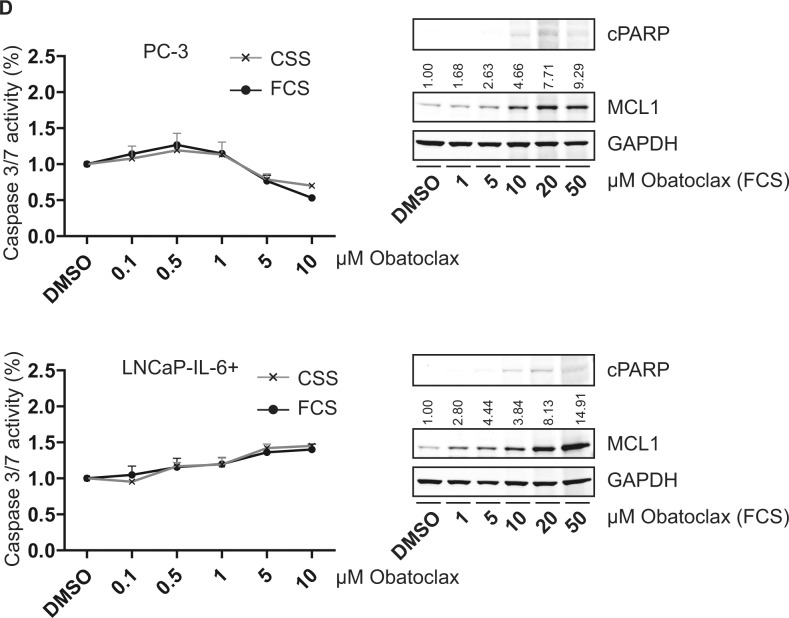
Obatoclax induces apoptosis in androgen-sensitive PCa cells but not in AR-negative PCa cells (A, B) Steroid-deprivation or the AR antagonist Bicalutamide tend to potentiate the pro-apoptotic effect of Obatoclax in androgen-sensitive LNCaP and DuCaP cells. (C) Progressed LNCaP-abl cells are insensitive to Obatoclax. (D) Castration-resistant, AR-negative PC-3 and LNCaP-IL-6+ cells are resistant to apoptosis induction by Obatoclax and react to Obatoclax treatment by upregulating MCL1. (A, C-D) PCa cell lines were steroid-deprived for ≥48 h using medium supplemented with 10% CSS and then treated for 24 h with increasing concentrations of Obatoclax or DMSO in medium containing 10% FCS or 10% CSS, as indicated. B) LNCaP and DuCaP were steroid-deprived for ≥48 h using medium supplemented with 10% CSS and then treated for 24 h with increasing concentrations of Obatoclax or DMSO in medium containing 10% CSS with the addition of 1 nM R1881 and 10 μM Bicalutamide (Bic) or vehicle (DMSO), as indicated. (A-D) Graphs show relative activity of cleaved Caspase 3/7 normalized to total protein. Additionally, immunoblotting was performed using antibodies detecting MCL1, cPARP and the reference protein GAPDH. Representative immunoblots are shown. Numbers indicate relative ratios of densitometrical analysis of MCL1 divided by the reference protein GAPDH. Significant differences were calculated between samples treated in medium supplemented with 10% FCS versus 10% CSS and samples treated in medium supplemented with 10% CSS and 1 nM R1881 and 10 μM Bicalutamide versus 10% CSS and 1 nM R1881 and vehicle (DMSO). *p<0.05; ***p<0.001.

Based on the concept that MCL1 protects cells from undergoing apoptosis during steroid-deprived conditions, we compared the cell death inducing properties of the BH3-mimetic Obatoclax targeting MCL1 [10] under various conditions: a) full growth (10% FCS), b) steroid-deprived (10% CSS), c) activation of AR signaling (10% CSS + 1 nM R1881), d) inactivation of AR signaling by the antagonist Bicalutamide (10% CSS + 1 nM R1881 + 10 μM Bic). When Obatoclax was tested on LNCaP and DuCaP cells, a concentration-dependent decrease of MCL1 expression levels was observed (Fig. 5A). As a consequence, apoptosis as evidenced by cPARP in immunoblots and cleaved caspase 3/7 activity assays, was increased. When tested under steroid-deprived conditions, DuCaP cells were significantly more susceptible for apoptosis induction by Obatoclax compared to full growth conditions and the same trend could be observed in LNCaP. Moreover, when tested under conditions where AR was activated or inactivated through R1881 or R1881 and Bicalutamide, respectively, Obatoclax showed higher efficiency when AR activity was blocked (Fig. 5B). The same experiments were repeated with the LNCaP-abl progression model (Fig. 5C and S3A). Immunoblots showed that Obatoclax was still able to decrease MCL1 expression levels, however, no significant induction of apoptosis could be observed. Experiments with the AR-negative PC-3 and LNCaP-IL-6+ revealed that both castration-resistant cell lines were refractory to apoptotic induction by Obatoclax up to 10 μM (Fig. 5D and S3B). Only a mild induction of apoptosis was observable when Obatoclax concentration was increased up to 50 μM. Interestingly, a dose-dependent increase of MCL1 through Obatoclax treatment was detected. This suggests that MCL1 is a targetable molecule in early stage PCa.

### Obatoclax inhibits clonogenic potential of primary basal tnPCa cells

**Figure 6:**
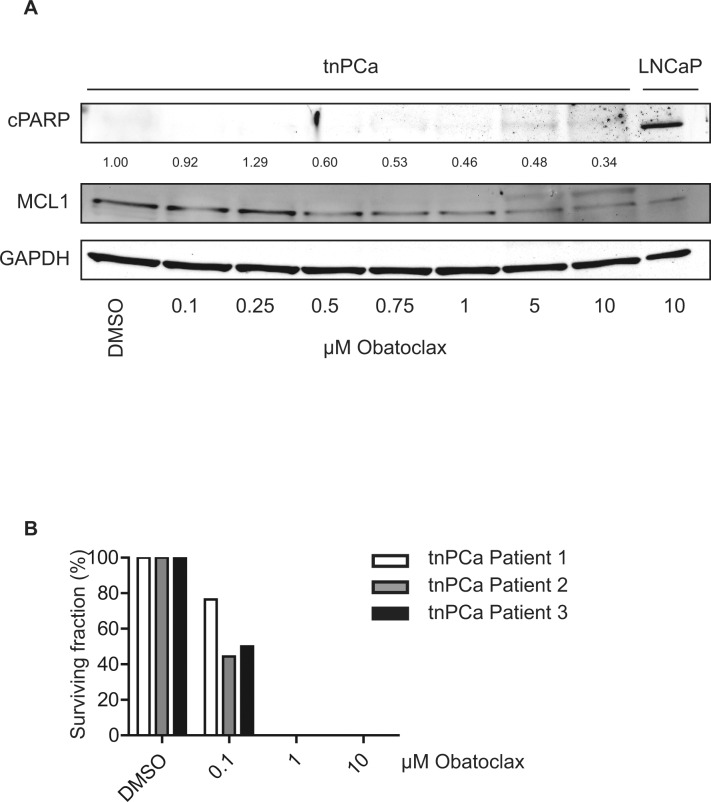
Obatoclax does not induce apoptosis in primary proliferating prostate cancer cells, while it is a potent inhibitor of clonogenicity (A) Proliferating primary basal tnPCa cells are insensitive to Obatoclax treatment. Primary basal tnPCa cells were kept under proliferating conditions and incubated with increasing concentrations of Obatoclax for 24 h. Immunoblotting was performed using antibodies detecting MCL1, cPARP and the reference protein GAPDH. A representative immunoblot from one out of three patients is shown. Numbers indicate relative ratios of densitometrical analysis of MCL1 divided by the reference protein GAPDH. (B) Obatoclax inhibits clonogenic growth of primary basal tnPCa cells. Primary basal tnPCa cells from three patients were seeded at low density (30 cells/cm²) and treated in growth medium with increasing concentrations of Obatoclax or vehicle (DMSO) for 12 days. Mean colony number was calculated and depicted as relative surviving fraction for each patient.

Next, we tested whether primary basal, androgen-independent tnPCa cells would be sensitive to Obatoclax treatment. As previously found in cell lines (Fig. 5A, B), Obatoclax treatment led to a dose-dependent decrease of MCL1 expression levels, when cells were kept under full growth conditions (Fig. 6A). However, induction of apoptosis as evidenced by cPARP was not observable. Since increased MCL1 mRNA levels could be seen in TIC compared to benign SC (Fig. 1C), we assessed the reproductive integrity of tnPCa cells using clonogenic assays (Fig. 6B). When cells were seeded at low density, a decreased colony number was counted upon incubation with 0.1 μM Obatoclax, while 1 μM was sufficient to completely abolish clonogenic growth. This shows that Obatoclax is effective in inhibiting the reproductive integrity of primary basal tnPCa cells.

## DISCUSSION

MCL1 has been proposed to have a unique role among the pro-survival members of the BCL2 family [5] that protect cells, and in particular cancer cells of diverse origins, against various anti-cancer treatments. For instance, high MCL1 expression in multiple myeloma was correlated with recurrence after chemotherapy and shorter survival [22] and mice bearing MCL1 overexpressing lymphomas were less sensitive to cyclophosphamide treatment *in vivo* [23]. Previously, we detected increased expression of MCL1 in docetaxel (Taxotere^TM^)-resistant PCa cell lines, as well as in tissue derived from PCa patients after neoadjuvant docetaxel treatment [24]. Similarly, cisplatin-resistant cell lines of various tissue origins showed high expression of MCL1 compared to their sensitive counterparts, while BCL2 and BCL2L1 were up-regulated at low frequency [25]. This shows that MCL1 is a major mediator of resistance against chemotherapy. Here, we found that MCL1 is also protecting against the cell death-inducing effects of endocrine therapy. In fact, ADT-mediated G_1_ cell cycle arrest could be interpreted as a partial resistance mechanism to this therapy. Importantly, only surviving cells (i.e. complete ADT-unresponsive or ADT-cell cycle arrested cells) can be at the basis of a progression to a castration-resistant disease. In consequence, targeting MCL1 concurrent with an induction of apoptosis in the ADT-mediated cell cycle-arrested PCa cell population could prevent or delay development of castration-resistance.

The AR signaling axis and cell cycle progression are two closely interlinked cellular processes that mutually regulate each other [2]. After AR inactivation through steroid deprivation, increased expression of MCL1 was detectable in LNCaP, a cell line that reacts with G_1_ cell cycle arrest to AR inhibition [16,26,27]. On the other hand, a role for MCL1 as interacting partner of proliferating cell nuclear antigen (PCNA) was proposed to inhibit cell cycle progression in S phase [28]. During prolonged mitotic arrest MCL1 was found to be degraded and to induce apoptosis [21]. Furthermore, MCL1 promoted terminal mitosis and differentiation involving p27^KIP1^ [29]. These examples show that the mutual interactions of AR/cell cycle/MCL1 signaling pathways in prostate cells result in a highly complex network. It is however unclear whether MCL1 is a regulator of cell cycle or whether MCL1 expression is regulated by cell cycle progression.

Pharmacological targeting of MCL1 by Obatoclax was efficient in androgen withdrawal-sensitive LNCaP and DuCaP, but inefficient in androgen withdrawal-insensitive cells such as LNCaP-abl, PC-3 and LNCaP-IL-6+. This loss of cell death-inducing activity of Obatoclax could be due to decreased/absent expression of pro-apoptotic BAK1 and BAX, which was previously demonstrated in PC-3 cells [30]. Similar to our results in PCa cells, it was found that endoplasmic reticulum stress induced up-regulation of MCL1 and rendered melanoma cells more sensitive to Obatoclax treatment [31]. Furthermore, Obatoclax was able to induce apoptosis in primary chronic lymphocytic leukemia cells previously resistant to treatment with oncolytic vesicular stomatitis virus [32]. Sabutoclax, another BH3-mimetic targeting MCL1, could sensitize prostate cancer cells to IL-24 mediated cytotoxicity [33]. We deduce from these findings that MCL1 inhibition is acting as a secondary trigger to induce apoptosis in cells in which the primary treatment/trigger is insufficient to cause this effect. This might also explain the absent pro-apoptotic effect of Obatoclax on primary PCa cells (Fig. 6A), where Obatoclax was used as a single agent. Although the clinical development of Obatoclax has been discontinued during this study [34], our results may encourage further research into BCL2 family inhibitors, and in particular in small molecules targeting MCL1. For example, the recently identified hydroxyquinoline-derived compound 9, shows selective MCL1 inhibitory function [35]. Possible clinical studies could select patients based on results of a BH3 profiling assay, which measures dependency of any or all anti-apoptotic BCL2 proteins for cellular survival [35].

High MCL1 expression was found in various cancer types [36]. In this study, we found a high expression of MCL1 by immunohistochemistry in the tissue of tnPCa patients (Fig. 1A, B), although we could not confirm a correlation of MCL1 staining score with Gleason score, as previously published [37]. Analysis of larger cohorts could clarify this discrepancy. Possible mechanistic explanations for increased MCL1 expression may be an amplification of the *MCL1* gene locus, as detected in other cancer types [6] and cytokine and growth factor signaling resulting from tissue inflammation known to increase MCL1 expression [5]. Moreover, we detected also high MCL1 mRNA expression in the TIC populations from tnPCa samples (Fig. 1C). Similarly, MCL1 was found to be increasingly expressed in the side population of non-small cell lung cancer cell lines [38]. Intriguingly, inhibition of MCL1 by Obatoclax was able to suppress self-renewal of those cells, while we found that Obatoclax is also a potent inhibitor of PCa clonogenicity (Fig. 6B). In addition, overexpression of MCL1 was found to induce malignant transformation of hematopoietic stem cells [23]. In summary, this shows that MCL1 is increasingly expressed and plays a role in the fate of cells in several compartments within PCa tissue and indeed that it could play an important role in PCa tumor initiation.

**Figure 7:**
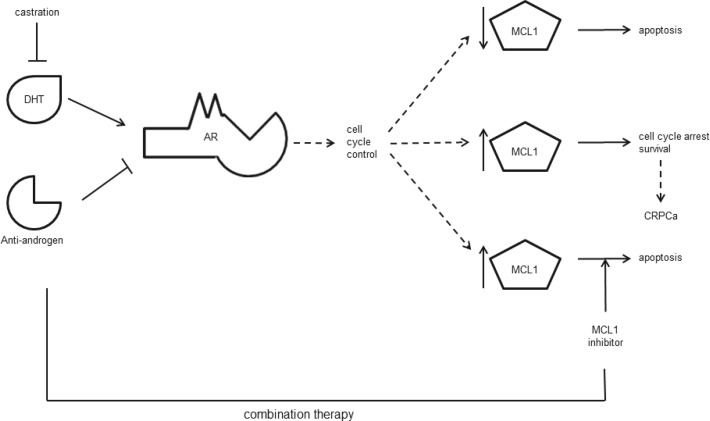
Schematic conclusion AR activity may be inhibited by androgen withdrawal (castration) and/or anti-androgens that compete with the natural ligand dihydroxytestosterone (DHT) for binding. AR is an important controller of cell cycle progression. Upon AR inactivation, PCa cells undergo apoptosis (upper arrow) or induce a G_1_ cell cycle arrest concurrent with cell survival (middle arrow) and possible progression to CRPCa. Our data indicates that high expression of MCL1 protects PCa cells from undergoing apoptosis under these conditions. Thus, combination therapies targeting AR and MCL1 could improve the current ADT protocol (lower arrow).

In conclusion, we demonstrate that MCL1 is an important regulator of apoptosis in PCa cells. It is a mediator of immediate steroid-deprivation resistance in PCa cells that react with a G_1_ cell cycle arrest to AR inactivation. Hence, our data provides a mechanistic rationale for considering clinical studies, where MCL1 inhibiting therapies could be assessed for synergy with the existing endocrine therapy for locally advanced and metastatic, hormone-sensitive PCa patients (Fig. 7).

## MATERIALS AND METHODS

### Ethics statement

Investigation has been conducted in accordance with the ethical standards and according to the Declaration of Helsinki and according to national and international guidelines and has been approved by the authors’ institutional review board.

### Cell lines and culture

Human PCa cell lines LNCaP, VCaP and PC-3 were obtained from ATCC (LCG Standards, Wesel, Germany), DuCaP were obtained from Prof. J. Schalken (Center for Molecular Life Science, Nijmegen, The Netherlands) and were maintained as previously described [19]. The LNCaP sub-lines LNCaP-abl [16] and LNCaP-IL-6+ [17] were generated by long-term maintenance in medium containing 10% charcoal-stripped serum (CSS, Hyclone, THP, Vienna, Austria) or in full growth medium containing 5 ng/ml interleukin-6 (IL-6), respectively. The following cell lines are androgen-sensitive and express AR: LNCaP, LNCaP-abl, VCaP and DuCaP. High passages (>80) of LNCaP-IL-6+, as used in this study, and PC-3 are AR-negative and castration-resistant.

### Western blot

Immunoblotting was performed as previously described [39]. The following antibodies were used: α-MCL1 (1:500, S-19, Santa Cruz, Heidelberg, Germany); α-glyceraldehyde-3-phosphate dehydrogenase (1:50 000, GAPDH, Merck Millipore, Darmstadt, Germany); α-PARP p85 (cPARP, Promega, Mannheim, Germany).

### Quantitative Real-Time Polymerase Chain Reaction

Quantitative Real-Time Polymerase Chain Reaction (qRT-PCR) was performed as previously described [40] using Taqman Gene Expression assays for MCL1 (Product number: Hs01050896_m1), HPRT1 (Hs02800695_m1) and RPLP0 (Hs99999902_m1) (all from Life Technologies, Vienna).

### Caspase 3/7 activity assays

Activity of cleaved caspase 3/7 was measured as previously described [41] using the Caspase 3/7-Glo assay (Promega) and normalized to protein input determined by BCA assay (ThermoScientific, Vienna).

### Immunohistochemistry

Immunohistochemistry on a tissue micro array and on explanted xenografts was performed as previously described [24,42] using α-MCL1 (S-19, Santa Cruz).

### Primary basal cell culture

Primary basal cell culture was performed in York (results in Fig. 1) and Innsbruck (results in Fig. 6). Permission for the use of patient specimens has been given by the Ethical Committees of Medical University of Innsbruck (Nr.:UN4837:317/4.7) and University of York (LREC ref No: 07/H1304/121). In York, isolation of primary basal cells was performed as previously described [43]. In Innsbruck, the protocol had to be adapted to the quantity of available tissue material derived from 3 mm needle biopsies using the “outgrowth” method. In brief, tissue from cancerous areas was collected by a needle biopsy on fresh prostate tissue after prostatectomy. Malignancy was confirmed by the uropathologist (G.S.) on a section of the needle biopsy taken before *in vitro* culture by routine diagnostics (haematoxylin and eosin, and p63/alpha-methylacyl-CoA racemase stainings). Tissue was then cut into small wedges which were put into a collagen I-coated T25 flask (Corning, Wiesbaden, Germany). Wedges were kept in K-SFM medium (Life Technologies, Vienna) supplemented with 2.5 μg/500 ml EGF (Life Technologies), 25 mg/500 ml bovine pituitary extract (Life Technologies), 2 ng/ml Leukemia inhibitory factor (Merck Millipore, Darmstadt), 2 ng/ml stem cell factor (Merck Millipore), 100 ng/ml cholera toxin (SigmaAldrich, Vienna), 1 ng/ml Granulocyte macrophage colony stimulating factor (Merck Millipore) and a mixture of 1x antibiotics and –mycotics (Life Technologies) until basal epithelial cells started to grow out from the wedges. At this timepoint STO mouse embryonal fibroblasts (ATCC, LCG Standards) previously irradiated with a dosis of 60 Gy were added to epithelial cells to form a confluent feeder layer. After one week tissue wedges were removed and the described procedure with the removed wedges was repeated twice more. Epithelial cells were grown until 80% confluence and passaged by trypsinization or seeded into collagen I-coated (BD Biosciences, Heidelberg) 6-wells for experiments. Stem/Tumor-initiating (SC/TIC), transit amplifying (TA) and committed basal (CB) cells were isolated as previously described [43].

### Cell cycle analysis

The following treatments were performed to arrest cells in different cell cycle phases: a) cycling: normal growth conditions (medium containing 10% CSS for LNCaP-abl or medium containing 10% FCS for all other cell lines); b) CSS, G_1_: incubation with medium containing 10% CSS for 3 days; c) serum(−), G_1_: incubation with medium without addition of serum for 3 days; d) thymidine G_1_/S: incubation in normal growth medium and 10 mM thymidine for 3 days; e) nocodazole, G_2_/M: incubation in normal growth medium and 200 ng/ml nocodazole for 18 hours. Cells were then harvested using a cell scraper, fixed in 70:30 (v/v) ethanol:PBS at 4°C, and digested with 50 μg/ml RNase A (Roche, Vienna) for 30 min at room temperature. Cells were stained with 50 μg/ml propidium iodide in PBS. Cell cycle distribution was analyzed using the FL3-A channel of a FACSCalibur (BD Biosciences) and visualized using FCS Express Flow Cytometry 4 software (De Novo Software, Glendale, CA, USA). Doublets were discriminated by plotting FL3-A events against FL3-W and gating for singlets.

### Animal experiments

Animal protocols were approved by the Austrian Federal Ministry for Education, Science and Culture (BMWF-66.011/0130-II/10b/2009, BMWF-66.011/0116-II/3b/2011). Xenografted tumors were grown by subcutaneous implantation of a 0.1 ml suspension of 2×10^6^ LNCaP cells mixed with 0.1 mL matrigel (BD Biosciences) into both, the right and the left flanks of anesthetized male nude mice (BALB/c/nu/nu, 4-6 weeks old, Charles River Laboratories, Sulzfeld, Germany), respectively. When the tumors became palpable, mice were randomly divided into three different treatment groups: ODN_Ctrl (5 mg/kg body weight), ODN_AR (5 mg/kg body weight) and castration. Oligonucleotides (ODNs) with 2`-O-(2-methoxy)ethyl modifications at the 5` and 3`ends, respectively, and phosphorothioated internucleotide linkages were purchased from GenXpress (VWR, Vienna): ODN_AR 5′-*u*g*c*ugaagagtagc*a*g*u*g-3` and ODN_Ctrl 5′-*a*g*a*ggcttgcacag*t*g*c*a-3`(modified bases are indicated by an asterisk). Castration was performed by orchiectomy of anesthetized mice. ODNs were dissolved in 0.9% (w/v) sodium chloride solution and administered intraperitoneally at a final concentration of 5 mg/kg mouse three times in the first week and twice from week 2 to 4. Explanted tumors were subdivided into two pieces and either frozen in liquid nitrogen for Western blot analysis or fixed in buffered formalin (4.5%) and embedded in paraffin for further immunohistochemical staining and qRT-PCR. LNCaP-abl cells (2×10^6^ cells mixed with matrigel in a 1:1 ratio) were injected into mice that received orchiectomy one week before.

### Clonogenic assay

Primary basal cells were trypsinized, counted and plated in full growth medium at a density of 30 cells/cm² (equals 300 cells per 6-well) in triplicates. Irradiated STO feeder cells were added to form a confluent layer (as described above). Treatment with Obatoclax (0.1 – 10 μM) or DMSO (vehicle) was performed on the day after seeding the cells. Medium was renewed every 2-3 days with new addition of Obatoclax and STO feeder cells. Colonies were stained as previously described [42]. Plating efficiency in DMSO treated samples was calculated by the number of colonies divided by number of cells seeded. Survival fraction was calculated by dividing the number of colonies by the product of plating efficiency and cells seeded.

### Chemicals

The synthetic androgen R1881 (methyltrienolone) was purchased from Organon (MSD, Vienna) and dissolved in ethanol at stock concentrations of 0.1, 1, and 10 μM. Bicalutamide (Casodex^TM^) was obtained from AstraZeneca (Macclesfield, UK) and dissolved in DMSO at a stock concentration of 10 mM. Obatoclax mesylate was purchased from Selleckchem.com (Eubio, Vienna) and dissolved in DMSO at a stock concentration of 10 mM.

### Chromatin immunoprecipitation

DuCaP and LNCaP were cultured for two days under steroid deprived conditions using medium containing 10% CSS. Cells were then treated in medium containing 10% CSS with addition of 1 nM R1881 or vehicle (EtOH). For chromatin immunoprecipitation the ChIPAb+E2F-1 kit from Merck Millipore was used following the manufacturer's instructions. QRT-PCR was performed on immunoprecipitated DNA and inputs using the following primer pairs: E2F-1 binding site on Mcl-1 promoter: forward 5′-cgc ccc ttt ccc ctt tta tgg-3′ and reverse 5′-gaa gac ccc gac tcc tta ctg g-3′ (derived from [20]). CDC2 primers were provided with the kit. Normalization was done using the formula 2^−dCt^ where dCt was calculated as the difference between Ct(immunoprecipitation) and Ct(input).

### Transfections, lentiviral infections and plasmids

PCa cell lines were transfected using Lipofectamine 2000 (Life Technologies) as previously described [41]. For downregulation of CDC20, FBXW7 and HUWE1 ON-TARGET plus smart pools consisting of four specific siRNA sequences and non-targeting control ON-TARGET plus smart pool (Dharmacon, ThermoScientific) were used and cells were transfected at a concentration of 50 nM. AR was downregulated by transfection of 30 nM with the following annealed constructs: sense 5′-GCACUGCUACUCUUCAGCAdTdT-3′ and antisense 5′-UGCUGAAGAGUAGCAGUGCdTdT-3′, as previously described [44].

The lentiviral plasmid for conditional MCL1 silencing was generated by GATEWAY-based recombination of sequence verified pENTR-THT-MCL1 [45] (targeting sequence 5′-CCATTAGCAGAAAGTATCA-3′ cloned into pENTR-THT-I) with pGLTR-X [46]. The generation of lentiviral plasmid for conditional MCL1 (pHR-tetCMV-hMCL-ires-GFP) was described elsewhere [45]. pHR-SFFV-rtTA-M2-T2A-Puro was made by amplifying rtTA2-M2 from pLIB-rtTA2-M2-ires-Puro [47] introducing a T2A sequence by T2A-sequence modified antisense primer. In a second reaction the puromycin N-acetyl transferase (pac) gene [48] was amplified using T2A-sequence modified sense primer. The PCR-products of both reactions were mixed and used as template for the third reaction using the rtTA2-M2 sense- and the puromycin antisense primer. The final 1.52 kb PCR product was purified, digested with BamHI and NotI and cloned into the BamHI/NotI site of pHR-SIN-CSGW (kindly provided by Mary Collins, UCL, London, UK) exchanging the eGFP with the rtTA2-M2-T2A-Puro cassette. The sequence verified plasmid was used to generate lentiviral particles.

Lentiviral infection of target cells was performed as described previously [45]. In brief, confluent HEK293T were transfected with 1.5 μg lentiviral vector, 0.9 μg pSPAX2 packaging and 0.9 μg pMD-G VSV-G-pseudotyping plasmids (both vectors were kindly provided by D. Trono, EPFL, Lausanne, Switzerland) by calcium phosphate-based transfection. Target cells were infected using 0.45 μm filtered virus containing cell culture supernatant obtained at 48 and 72 hours after transfection and supplemented with 1μg/ml polybrene (SigmaAldrich). For conditional overexpression, cells were infected with lentiviral vectors encoding sequences for rtTA2-M2, followed by selection of puromycin (1μg/ml). Puromycin resistant cells were superinfected with pHR-tetCMV-hMCL1-ires-GFP [45].

### Statistical analysis

The statistical significances of differences between treatment and control samples were calculated with Student's t-test using Graph Pad Prism. Comparison groups are given in the figures and/or figure legends and significances are encoded as follows: *p<0.05; **p<0.01; ***p<0.001. Statistical significances of differences in Fig. 1B and 2D have been calculated with Mann-Whitney U-test using Graph Pad Prism and p values are indicated in the figure or figure legend.

## SUPPLEMENTARY MATERIAL AND FIGURES


