# Supplementary material for: Mechanistic rationale for MCL1 inhibition during androgen deprivation therapy
Source: Oncotarget. 2015 Jan 14;6(8):6105–22. doi: 10.18632/oncotarget.3368 (PMC4467425; doi:10.18632/oncotarget.3368)
Supplement: Supplementary file 1 [file oncotarget-06-6105-s001.pdf]

# Mechanistic rationale for MCL1 inhibition during androgen deprivation therapy

## Supplementary Material

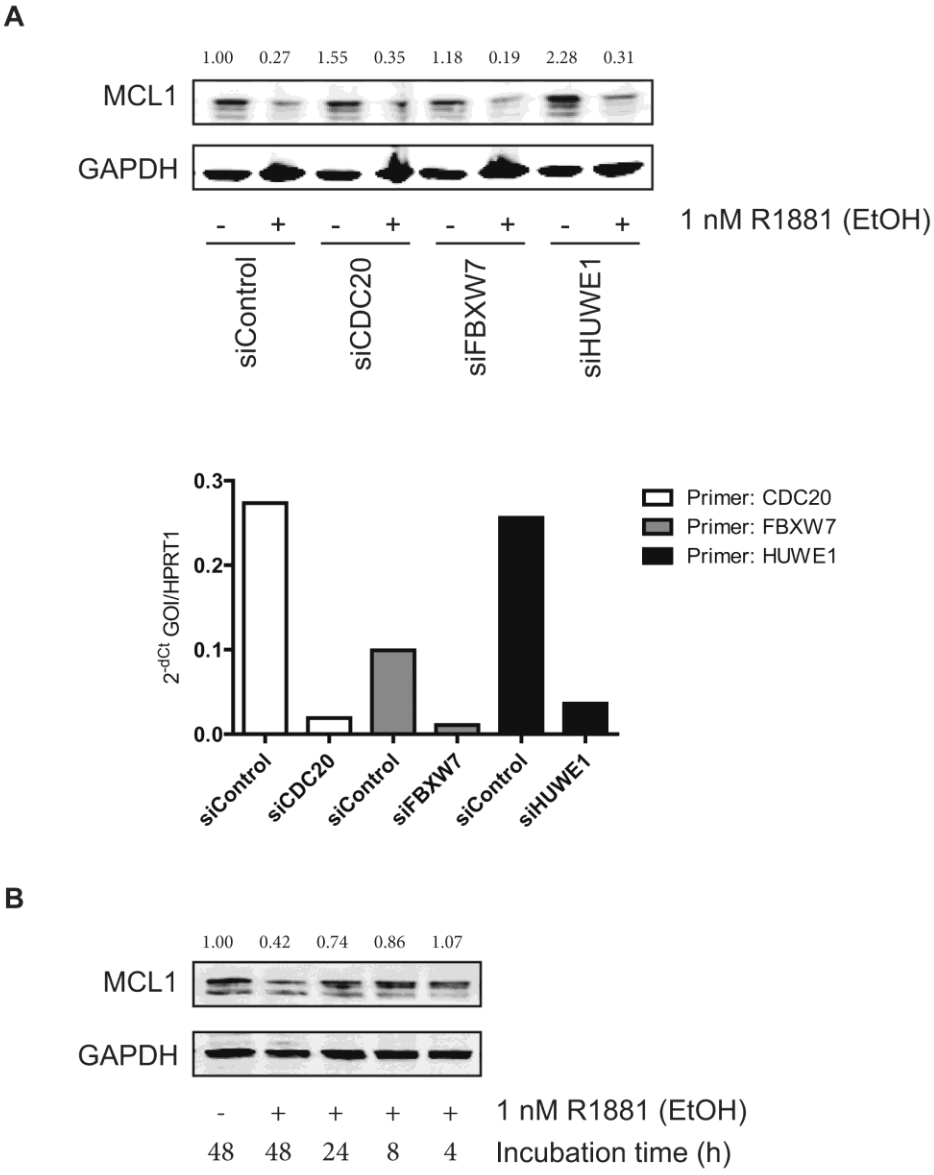


**Supplementary Figure S1: Androgenic regulation of MCL1 is dependent on functional AR and is a transcriptional mechanism.**

**(A) Androgenic repression of MCL1 is not mediated via E3-Ligases APC/C$^{CDC20}$, SCF$^{FBXW7}$ or HUWE1.** LNCaP cells were steroid-deprived for ≥48 h using medium supplemented with 10% CSS. After transfection with 50 nM siRNA targeting CDC20, FBXW7, HUWE1 or non-targeting siRNA (siControl), cells were treated with medium supplemented with 10% CSS and 1 nM R1881 or vehicle (EtOH), as indicated. Efficient knockdown of CDC20,

FBXW7 and HUWE1 was controlled by qRT-PCR using respective primers and values were normalized to the housekeeper HPRT1. **(B) Androgenic regulation of MCL1 protein is significant after 24 h of incubation with 1 nM R1881.** VCaP cells were steroid-deprived for ≥48 h using medium supplemented with 10% CSS and then treated with medium supplemented with 10% CSS and 1 nM R1881 or vehicle (EtOH) for different time points, as indicated. **(A, B)** Representative immunoblots using antibodies detecting MCL1 and the reference protein GAPDH are shown. Numbers indicate relative ratios of densitometrical analysis of MCL1 divided by the reference protein GAPDH.

A

| Gene | Chromosome:Start..End | Fold |
|---|---|---|
| BCL2L1 | Chr20:29715361..29716168 | 49,93 |
| BCL2L1 | Chr20:29763961..29764411 | 49,11 |
| BCL2L1 | Chr20:29731431..29731718 | 22,09 |
| BCL2L1 | Chr20:29762827..29763014 | 7,87 |
| BCL2L11 | Chr2:111628479..111629082 | 32,74 |
| BCL2L11 | Chr2:111659550..111659982 | 13,85 |
| BCL2L11 | Chr2:111721854..111722017 | 16,37 |
| BCL2L11 | Chr2:111629639..111629957 | 10,81 |
| BCL2L11 | Chr2:111718984..111719149 | 15,09 |
| BCL2L11 | Chr2:111619008..111619241 | 13,64 |
| BCL2L13 | Chr22:16500650..16501270 | 30,4 |
| BCL2L13 | Chr22:16510189..16510572 | 42,57 |
| BCL2L13 | Chr22:16500400..16500638 | 8,19 |
| BAK1 | Chr6:33632665..33632969 | 20,01 |
| BAK1 | Chr6:33647839..33648095 | 10,42 |
| BNIP3 | Chr10:133661060..133661606 | 38,2 |
| BNIP3 | Chr10:133661693..133661869 | 16,37 |

B

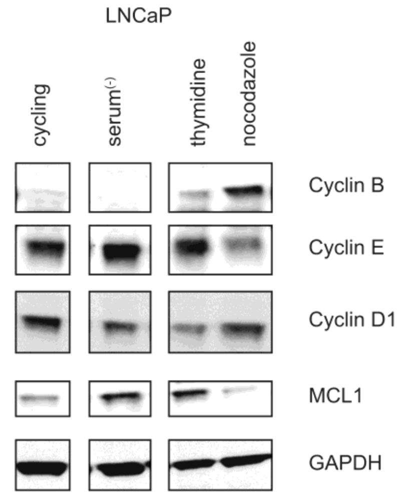


**Supplementary Figure S2: MCL1 is a cell cycle regulated protein.**

**(A) AR binding sites within loci of the BCL2 family.** Chromatin immunoprecipitation was performed on DuCaP cells treated for 1 h with 1 nM R1881 or vehicle (EtOH) using anti-AR antibodies. DNA bound on the precipitated AR complex was subjected to deep sequencing (ChIP-seq). The resulting gene list was searched for members of the BCL2 family. All identified sequences with the respective loci aligned to the unmasked human reference genome (NCBI v36, hg18) are depicted in the table. The fold change indicates the enrichment of AR bound to the respective sequence of R1881-treated versus vehicle-treated samples. **(B) Expression of cyclins after $G_1$, $G_1$/S, and $G_2$/M cell cycle arrest.** Successful cell cycle arrest was controlled by immunoblotting for cyclins B, E, and D1 in LNCaP cells after serum deprivation, thymidine excess, and nocodazole treatment, as described in Material and Methods. Additionally, MCL1 and the reference protein GAPDH are shown.

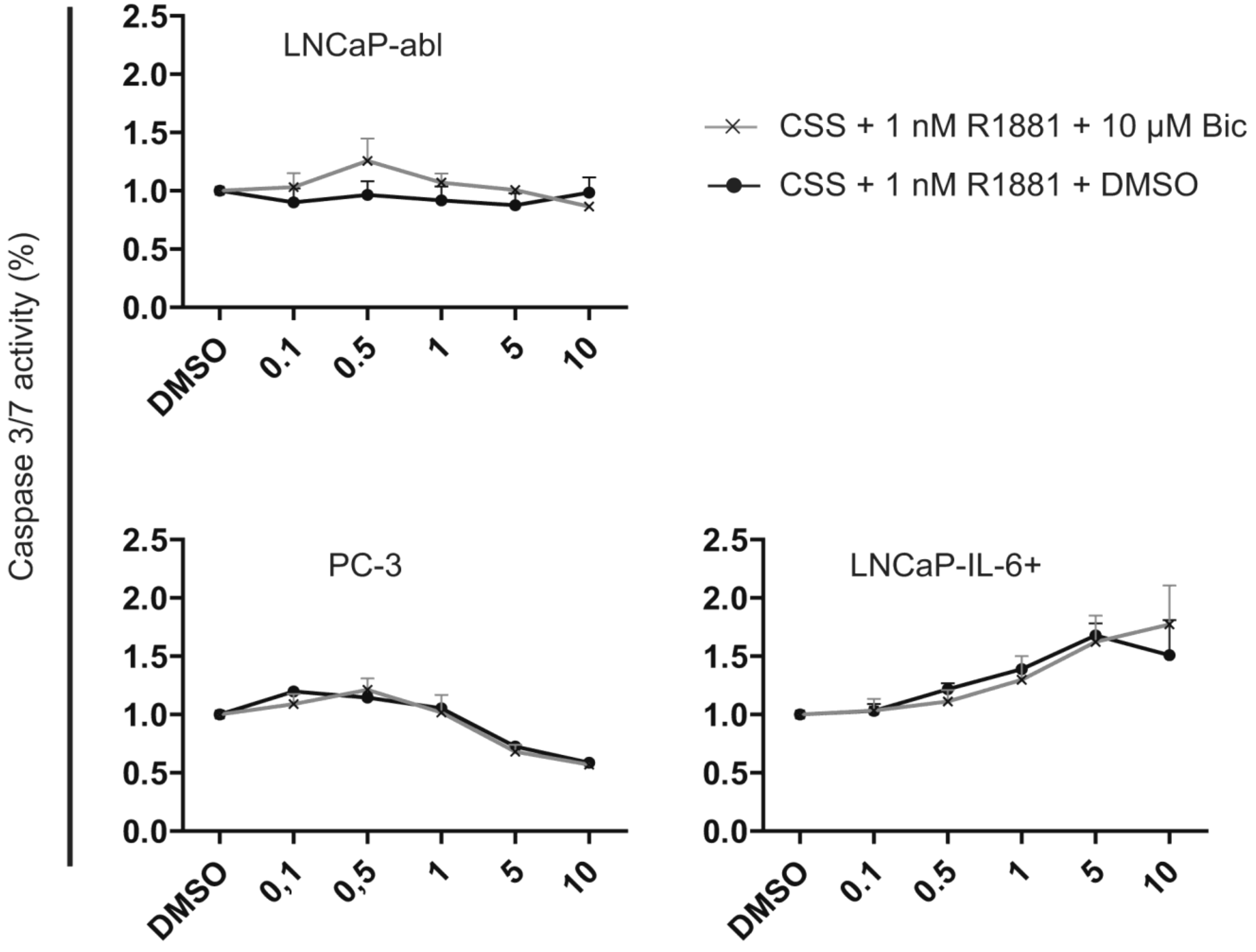


**Supplementary Figure S3: Obatoclax induces apoptosis in androgen-sensitive PCa cells but not in AR-negative PCa cells.**

PCa cell lines were steroid-deprived for ≥48 h using medium supplemented with 10% CSS and then treated for 24 h with increasing concentrations of Obatoclax or DMSO in medium containing 10% CSS and 1 nM R1881, as well as 10 µM Bicalutamide (Bic) or vehicle (DMSO), as indicated. Graphs show relative activity of cleaved Caspase 3/7 normalized to total protein input.
